# Supplementary material for: Experimental and Theoretical Study on Crown Ether-Appended-Fe(III) Porphyrin Complexes and Catalytic Oxidation Cyclohexene with O2
Source: Molecules. 2023 Apr 13;28(8):3452. doi: 10.3390/molecules28083452 (PMC10146806; doi:10.3390/molecules28083452)
Supplement: Supplementary file 1 [file molecules-28-03452-s001.zip › molecules-2315247-supplementary.pdf]

Experimental and theoretical study on crown ether-appended-Fe(III) porphyrin complexes and catalytic oxidation cyclohexene with O_2

Xiaodong Li[a], Ailing Feng*[a], Yanqing Zu[a], Peitao Liu[a], Fengbo Han[a], Meimei An[b]

[a] Institute of Physics & Optoelectronics Technology, Baoji University of Arts and Sciences, Baoji, 721016, China

[b] College of History Culture and Tourism, Baoji University of Arts and Sciences, Baoji, 721016, China

2. Experimental

2.1. Materials and Catalytic Property Studies

Scheme S1 shows the schematic diagram of the catalytic molecular oxygen oxidation device. To begin the experiment, a quantitative amount of substrate and catalyst is added to the 25mL branch flask (reaction flask), which is connected to the three-way valve. The flask is also connected to a gas volumeter with a readable volume of 50 mL through a condensing tube and a washing cylinder. The gas volumeter is also connected to a gas storage cylinder through a tee pipe. To eliminate air from the reaction system, the tee pipe is closed, and the piston of the flask branch pipe is opened, allowing oxygen from the meter to fill the reaction flask. This process is repeated three times. Once the air is removed, the piston of the branch tube is closed, and the reaction flask is only connected to the gas meter. The reaction flask is placed in a heating bath at a constant temperature, and the oxidation reaction is carried out with magnetic stirring. The change in the volume of oxygen in the meter is monitored regularly. After the reaction, the product is analyzed by GC. A blank test is performed to ensure the accuracy of the oxygen consumption measurement.

* Corresponding author at: Institute of Physics & Optoelectronics Technology, Baoji University of Arts and Sciences, Baoji, 721016, China.
E-mail address: ailingfeng@bjwlxy.edu.cn (A. Feng).

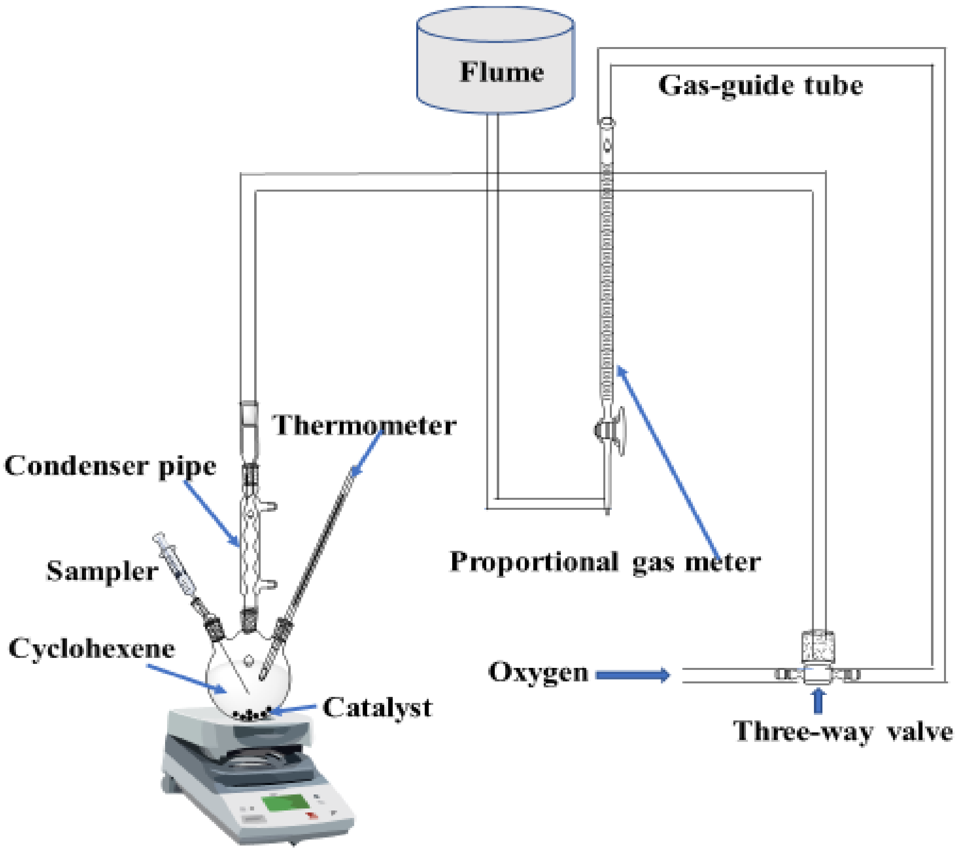

Scheme S1 Experimental schematic diagram of the catalytic oxidation reaction

2.2. Computational details

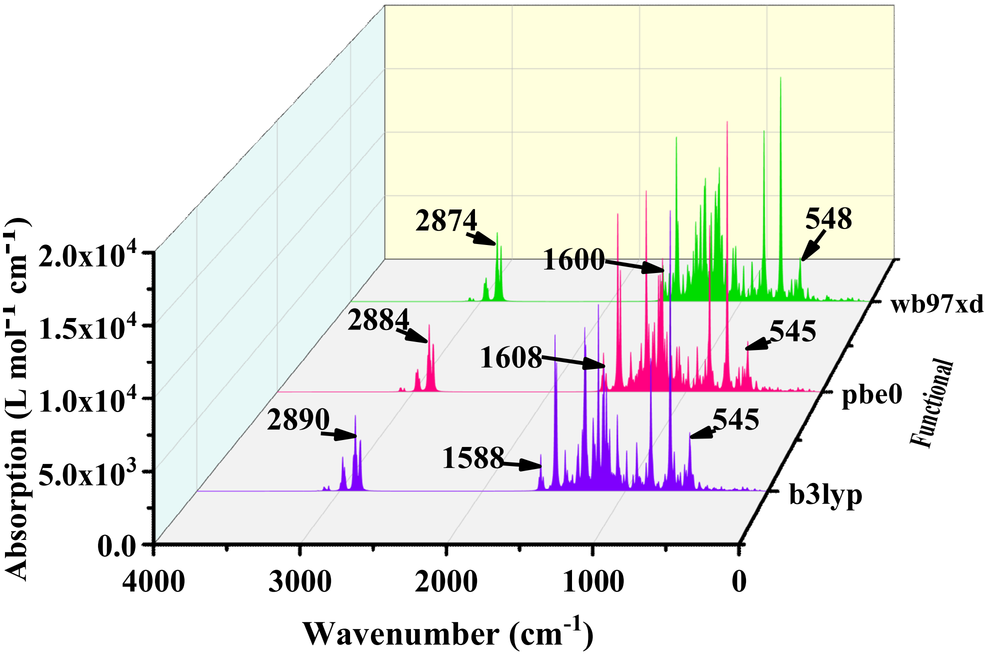

Fig. S1 Simulated infrared spectrum of FeTC4PCl. The frequencies have been scaled by the fundamental scaling factor.

3.5. Optimized structure and bonding properties

Table S1 The selected bond length of FeTPPCl, $FeTC_4PCl$ and $(Fe\text{-}O_2)TC_4PCl$ complexes calculated at ωB97XD/def2-SVP level of theory

Complexes	Distance (Å)				Angle (°)			
	N-Fe	Fe-Cl	Fe-O	O-O	Cl-Fe-O	Fe-O-O	C_1-N-N_p-C_{p1}	C_2-N-N_p-C_{p2}
FeTPPCl	2.09	2.20	-	-	-	-	-	-
$FeTC_4PCl$	2.09	2.20	-	-	-	-		
$Fe\text{-}O_2TPPCl$	2.00	2.25	2.98	1.99	172.72	118.28	164.38	167.59
$Fe\text{-}O_2TC_4PCl$	2.00	2.24	2.99	1.99	172.99	118.45	151.18	150.15

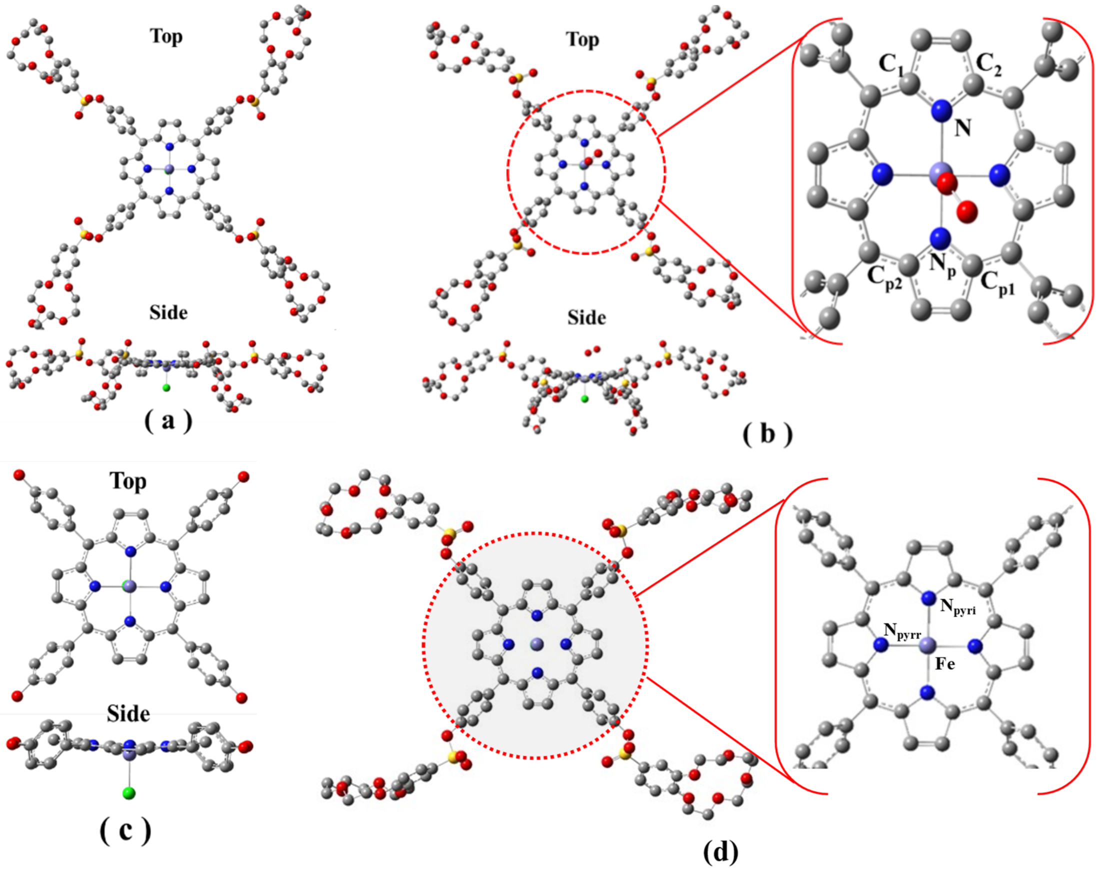

Fig S2. Optimized geometry of (a) $FeTC_4PCl$, (b) $(Fe\text{-}O_2)TC_4PCl$, (c) FeTPPCl and $FeTC_4PCl$ using ωB97XD/def2-SVP level calculation. To FeTPPCl and $FeTC_4PCl$ using sextet ground spin state and $(Fe\text{-}O_2)TPPCl$ and $(Fe\text{-}O_2)TC_4PCl$ compounds using octet ground spin state. The H atom is hidden for clarity.

3.6. Atomic charge analysis

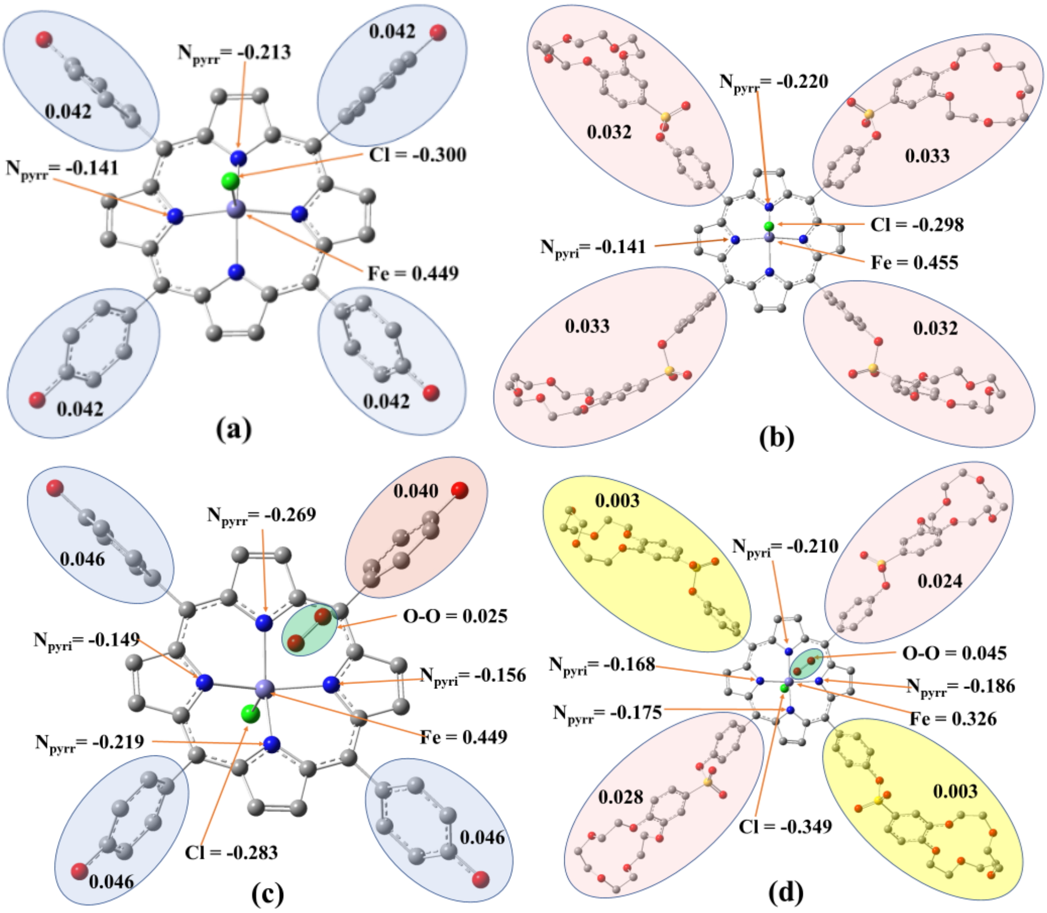

Fig S3 ADCH atomic charge analysis of (a) FeTPPCl, (b) FeTC4PCl, (c) (Fe-O_2)TPPCl, and (d) (Fe-O_2)TC_4PCl at ωB97XD/def2-SVP level of theory

3.7. Molecular orbital and spin density analysis

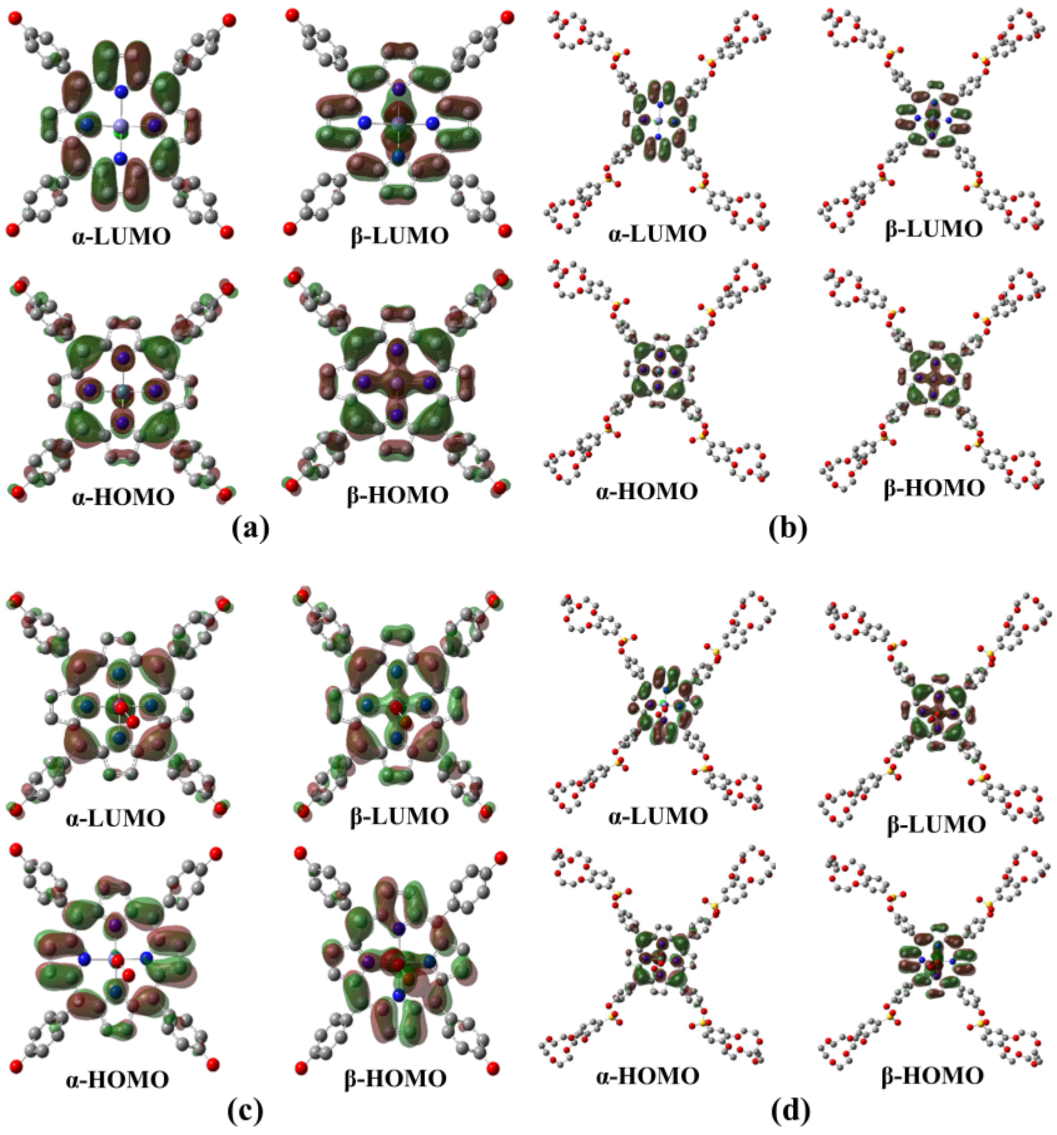

Fig S4. The frontier molecular orbital distributions of (a)FeTPPCl, (b) $FeTC_4PCl$, (c) $Fe\text{-}O_2TPPCl$, and (d) $Fe\text{-}O_2TC_4PCl$. (isovalue is 0.001 a.u)

3.7. Density-of-state analysis

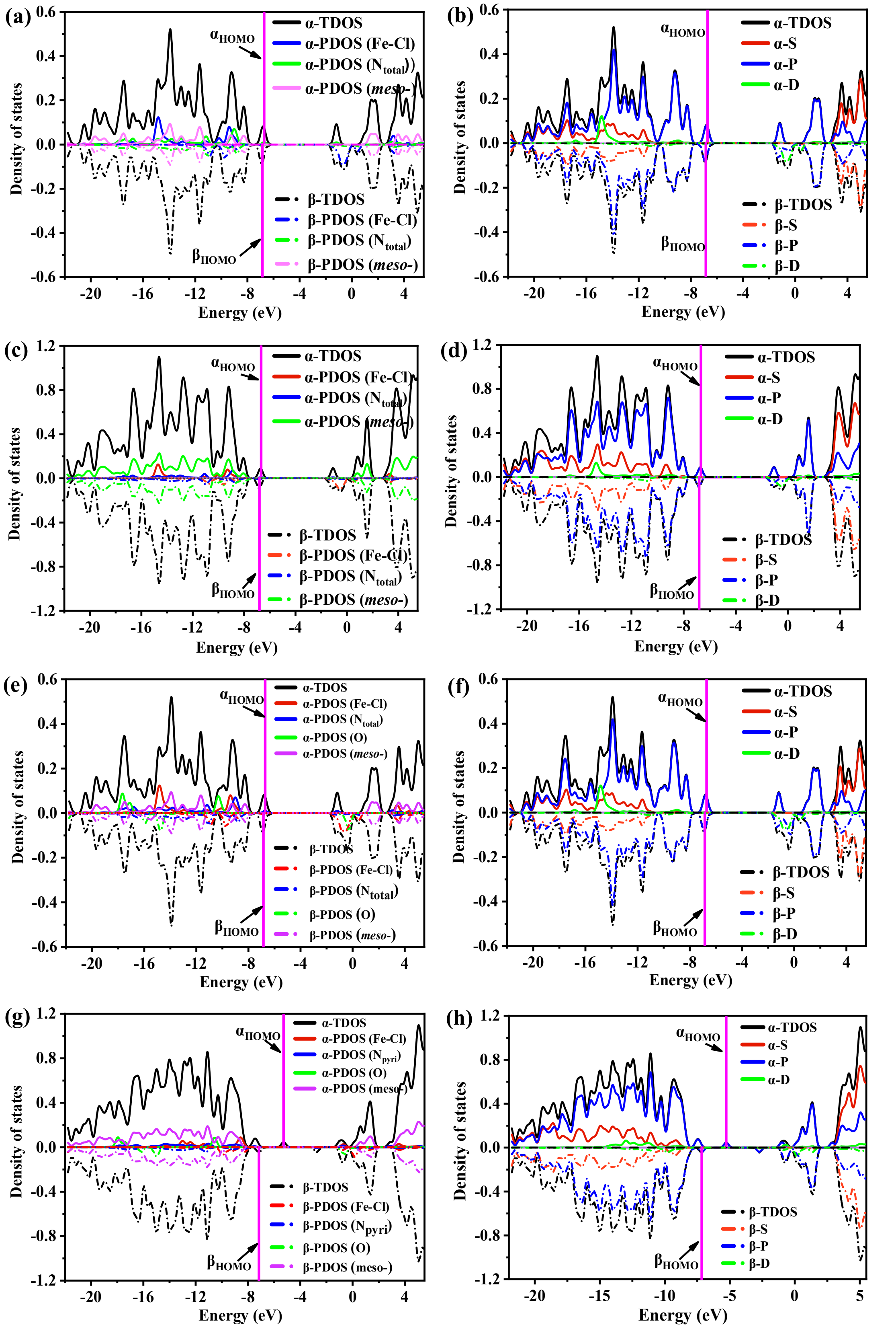

Fig S5. Density-of-state (DOS) map and MOs degeneracy of the (a, b) FeTPPCl, $FeTC_4PCl$ (c, d), (e, f) $(Fe\text{-}O_2)TPPCl$, and (g, h) $(Fe\text{-}O_2)TC_4PCl$ complexes. The location of the HOMO is carmine vertical line.
